# Diffusion-Weighted Magnetic Resonance Imaging in Suspicious Breast Lesions Detected on Ultrasonography and Mammography With Pathological Correlation

**DOI:** 10.7759/cureus.92094

**Published:** 2025-09-11

**Authors:** Amrita Das, Sujan Dibragede, Sukanya Goswami, Mary H Bhuyan, Punyada Padhy, Tanaya Sarma

**Affiliations:** 1 Department of Radiodiagnosis, Tezpur Medical College and Hospital, Tezpur, IND; 2 Department of Radiodiagnosis, Pragjyotishpur Medical College and Hospital, Guwahati, IND; 3 Department of Radiodiagnosis, Guwahati Medical College and Hospital, Guwahati, IND; 4 Department of Radiodiagnosis, Assam Medical College and Hospital, Dibrugarh, IND; 5 Department of Radiodiagnosis, Kalinga Institute of Medical Sciences, Odisha, IND

**Keywords:** adc, breast cancer, diffusion weighted, dwi mri, mammography, mri breast

## Abstract

Introduction

Diffusion-weighted magnetic resonance imaging (DW-MRI), an advanced functional imaging method, offers a notable advantage over traditional dynamic contrast-enhanced MRI (DCE-MRI) by generating tissue contrast without requiring the injection of intravenous contrast agents. The aim of the study is to evaluate the role of DW-MRI in suspicious breast lesions encountered primarily on ultrasonographic and mammographic evaluation.

Methods

A hospital-based cross-sectional study was performed between July 2023 and December 2023, including 34 suspicious breast lesions in 32 females detected on ultrasonography and mammography. Cases underwent MRI on a 1.5 Tesla scanner using a dedicated breast coil. DW MRI was done with b-values of 500, 800, and 1000 s/mm². Lesions were characterized by ultrasonography and mammography as per the ACR BI-RADS lexicon. Histopathological diagnosis was considered the gold standard of reference.

Results

Of 34 breast lesions, 16 (47.1%) were benign and 18 (52.9%) were malignant. The cut-off ADC value derived by the receiver operating characteristic (ROC) curve was 1.12 x10-3 mm²/s using different b values of 500, 800, and 1000 s/mm², which yielded optimal sensitivity (88.6%) and specificity (78.6%). The mean apparent diffusion coefficient (ADC) values of benign and malignant lesions were 1.51±0.22 x10-3 mm²/s and 1.0±0.09x10-3 mm²/s, respectively (p<0.0001). The mean ADC value of normal breast parenchyma was 1.68 ± 0.02 x 10-3 mm²/s (p<0.0001). DW‑MRI achieved a sensitivity, specificity, positive predictive value, negative predictive value, and accuracy of DW-MRI by using ADC mapping that were found to be 88.6%, 78.6%, 83.8%, 84.6%, and 84.1%, respectively, in this study.

Conclusion

DW-MRI can be a potential adjunct to routine screening and diagnostic modalities and can be used as a problem-solving tool in suspicious breast lesions encountered on conventional imaging.

## Introduction

Female breast cancer is the most prevalent cancer worldwide, with an estimated 2.3 million new cases in 2020 [1]. Identifying accurate screening and diagnostic tools is critical for effective disease management. Despite significant improvements in the use of conventional breast imaging modalities such as mammography and ultrasound, distinguishing between benign and malignant breast lesions remains a diagnostic challenge, especially in women with dense breast tissue [2].

Dynamic contrast-enhanced magnetic resonance imaging (DCE-MRI) is currently the most sensitive breast imaging modality and has demonstrated higher sensitivity than both mammography and ultrasound [3]. However, its high cost, time requirements, and reliance on contrast agents limit its utility as a routine screening tool. Additionally, concerns have emerged about the potential for gadolinium-based contrast agent deposition in the brain following repeated use.

As a result, there is a growing need for a non-contrast imaging technique that is both time- and cost-effective and that can complement conventional breast imaging [4]. In 1997, Englander et al. first explored the application of diffusion-weighted imaging (DWI) in the human breast. Since then, multiple studies have demonstrated DWI to be a highly sensitive tool in breast imaging [5].

However, the lack of standardization in imaging protocols and interpretation has hindered the integration of DWI into the MRI BI-RADS lexicon. Although the application of DWI in breast imaging remains under investigation, it holds promising potential to provide both qualitative and quantitative information about breast lesions. Therefore, the present study aims to evaluate the role of diffusion-based breast imaging.

## Materials and methods

This prospective observational study was conducted in the Department of Radiodiagnosis, Assam Medical College & Hospital, Dibrugarh, from July 2023 to December 2023. A total of 34 lesions in 32 female patients, aged between 35 and 63 years, were included. The inclusion criteria were (I) female patients aged 35 years and above and (II) patients with suspicious breast lesions on X-ray mammography and/or ultrasound (classified as ACR BI-RADS 4 or 5). Written informed consent was obtained from all patients prior to the procedure. The details of the procedure were explained in clear and easily understandable language to ensure patient comprehension. The higher BI-RADS category was assigned in cases where lesions had different classifications across imaging modalities to avoid underestimation. Exclusion criteria were (I) patients with contraindications to MRI (e.g., pacemakers, metallic implants), (II) purely cystic breast masses, (III) claustrophobic patients, (IV) pregnant women, and (V) male breast pathology. Patients with clinically diagnosed breast masses were prospectively recruited from various outpatient and inpatient services of the clinical departments.

MRI technique

MRI was performed using a 1.5 T magnet (Siemens Magnetom Avanto Fit 1.5T, A TIM + DOT whole-body MRI system; Siemens Medical Solutions, Munich, Germany) with a dedicated bilateral breast surface coil in the prone position. The present study included an unenhanced protocol including axial T1-weighted non-fat-suppressed, T2-weighted STIR, and echo-planar diffusion-weighted imaging (DWI). These patients underwent DCE-MRI breast in our department with the standard protocol. However, only the above-mentioned sequences were evaluated for the present study, which was taken prior to contrast administration.

The MR parameters used in DWI imaging (free-breathing diffusion-weighted single-shot single-echo-type echo-planar sequence fat suppression with spair was performed in the axial plane) are as follows: field of view (FOV) of 340 mm, slice thickness of 3 mm, rep time/echo time (TR/TE) of 5200/87 ms, flip angle of 170°, voxel size of 2.7 x 2.7 x 4.0 mm, matrix size of 128 x 96, b1 value of 500 s/mm², b2 value of 800 s/mm², and b3 value of 1000 s/mm². The diffusion gradient was applied sequentially in the three orthogonal directions. Apparent diffusion coefficient (ADC) maps were reconstructed from the diffusion-weighted images. For short tau inversion recovery (STIR) MR imaging, the following parameters are used: FOV of 340 mm, slice thickness of 3 mm, TR/TE of 4900/70 ms, and flip angle of 170°. For non-fat-suppressed fast low-angle shot (FLASH) T1-weighted images, whole breast transverse orientation was applied with the following parameters: FOV of 340 mm, TR/TE of 680/7.2 ms, and slice thickness of 3 mm.

Image Analysis

For qualitative characterization, following visual inspection of DWI images and ADC maps, the lesions were categorized according to the presence and absence of true restricted diffusion at various b-values. For quantitative assessment, the ADC maps were automatically generated by commercially available software. The ADC values of the lesions were calculated using the diffusion sensitizing gradients of b-500, b-800, and b-1000 sec/mm².

The placement of ROIs was done manually with the help of the unenhanced T1 sequences, T2 (STIR) sequences, and DWI sequences. ADCs were calculated automatically per pixel at a scanning console using b values of 500, 800, and 1000 s/mm², displayed as an ADC map. The mean ADC values of the lesions found on MRI were measured by using regions of interest (ROIs) within the targeted masses on ADC maps manually. Initially, a slice from the axial ADC maps representing the mass lesion with the largest diameter was selected. Then, multiple ROIs were placed (as many as possible) within the mass lesion by referring to DWI and the unenhanced sequences for verification of the lesion boundaries. The ROIs were carefully put inside the mass so that they did not run off the edge to avoid partial volume effects. The obvious cystic area and the visual artifacts of DWI were avoided. The cut-off mean ADC value for differentiating benign and malignant lesions was calculated using ROC curve analysis.

The ADC value of normal fibroglandular tissue was measured by placing ROI while trying to omit fatty tissue at one place in the contralateral or ipsilateral normal breast parenchyma. The relative ADC (rADC) was calculated as the mass ADC value divided by the ADC value of adjacent parenchyma.

Statistical Analysis

Statistical analysis was performed using IBM Corp. Released 2007. IBM SPSS Statistics for Windows, Version 16.0. Armonk, NY: IBM Corp. Receiver operating characteristic (ROC) curve analysis was done to take out the cut-off values of ADCs. Discrete data were expressed as numbers (%) and analyzed using Fisher’s exact test. Results on continuous measurements were presented as mean ± SD and compared using Student’s t-test. For all analyses, the statistical significance was fixed at the 5% level.

## Results

**Figure 1:**
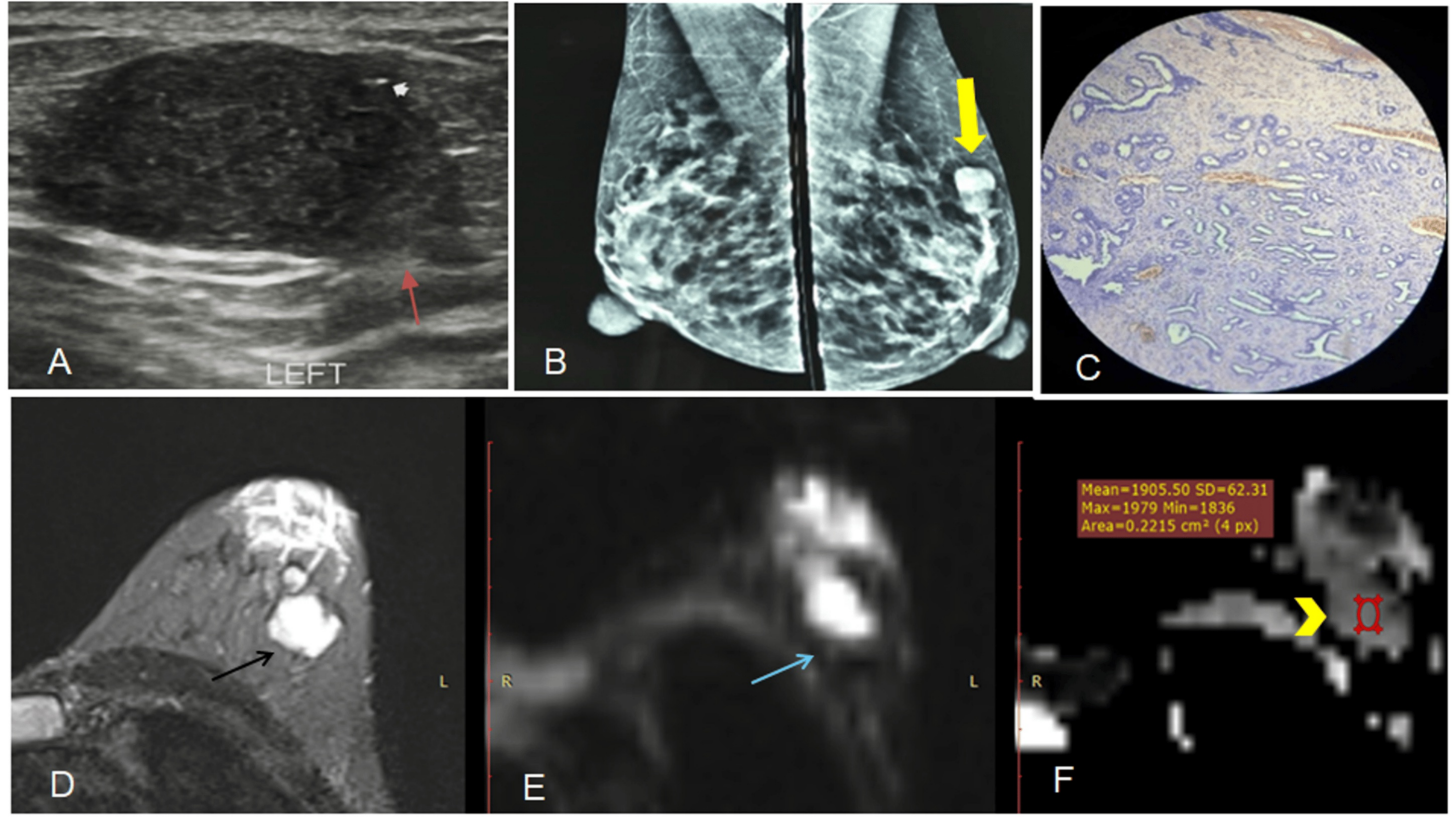
Fibroadenoma (A) Ultrasound shows an oval, hypoechoic lesion with intralesional calcification (white arrow) and indistinct margins (red arrow), categorized as BI-RADS IVa. (B) Mammogram (MLO view) reveals a circumscribed, irregular high-density lesion (yellow arrow) in the upper outer quadrant of the left breast, assessed as BI-RADS III. (C) Photomicrograph confirms the diagnosis of fibroadenoma (histopathological examination after excision). (D) Axial STIR image demonstrates a high signal intensity lesion (black arrow). (E) Axial DWI shows hyperintensity (blue arrow), (F) with the corresponding ADC map (b = 800) revealing a high ADC value (yellow arrowhead); [ADC = 1.2 × 10⁻³ mm²/s], consistent with benign etiology.

**Figure 2:**
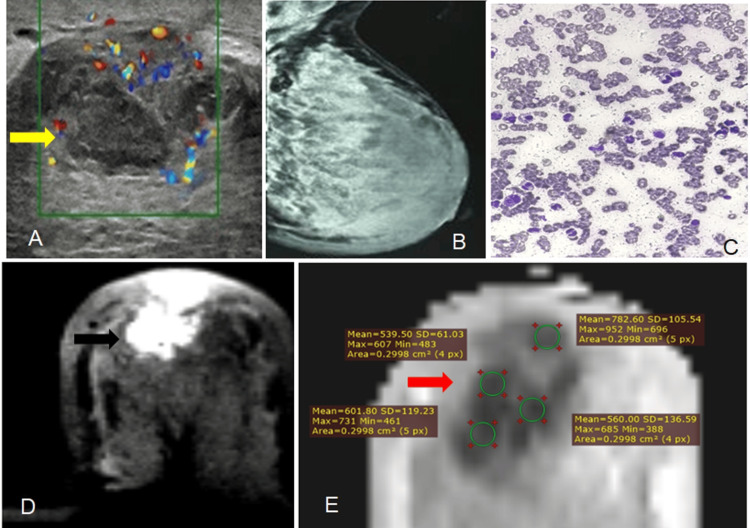
Mastitis (A) Ultrasound shows an irregular, vascular, heteroechoic space-occupying lesion (yellow arrow) with indistinct margins in a parallel orientation, surrounded by inflammatory changes, categorized as BI-RADS IVc. (B) Mammography (MLO view) shows a diffuse increase in breast density with thickening of the inferior medial skin fold; no discrete mass is identified [BI-RADS III]. (C) Fine-needle aspiration cytology (FNAC) shows a predominance of inflammatory cells, mainly polymorphs. (D) Axial DWI demonstrates marked hyperintensity (black arrow). (E) The corresponding ADC map (b = 1500) shows significantly restricted diffusion with a low ADC value (red arrow); [ADC ~ 0.52 × 10⁻³ mm²/s].

**Figure 3:**
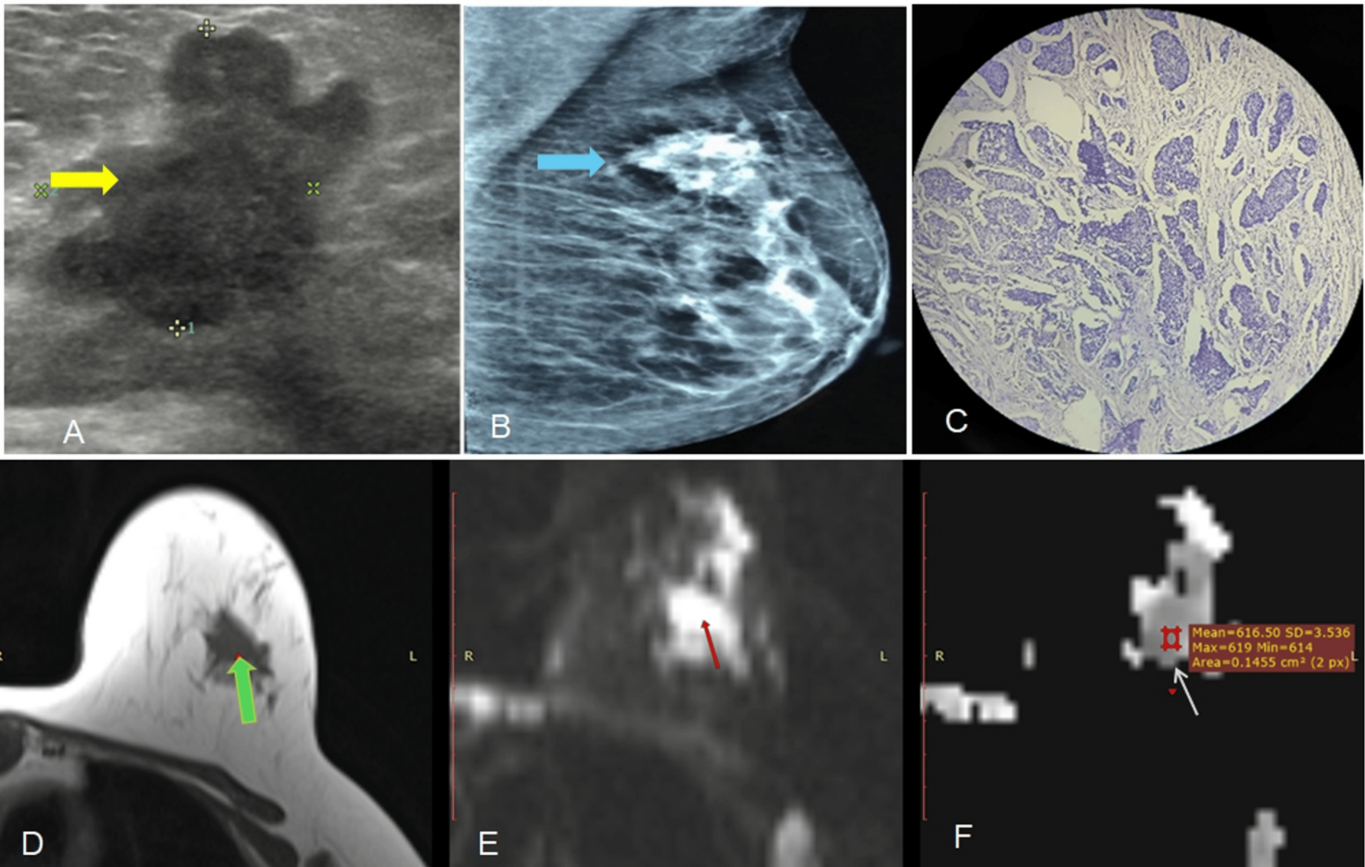
Invasive Ductal Carcinoma (A) Ultrasound demonstrates a hypoechoic, irregular lesion (yellow arrow) with microlobulated margins and non-parallel orientation, categorized as BI-RADS V. (B) Mammography (MLO view) reveals an irregular, high-density mass (blue arrow) with indistinct margins in the upper quadrant, assigned BI-RADS IVc. (C) Histopathological examination confirms invasive ductal carcinoma (IDC) on photomicrograph. (D) Axial T1-weighted MRI shows a hypointense lesion (green arrow). (E) Axial DWI reveals restricted diffusion (red arrow). (F) The corresponding ADC map (b = 1000) shows low ADC values (white arrow); [ADC = 0.62 × 10⁻³ mm²/s].

**Figure 4:**
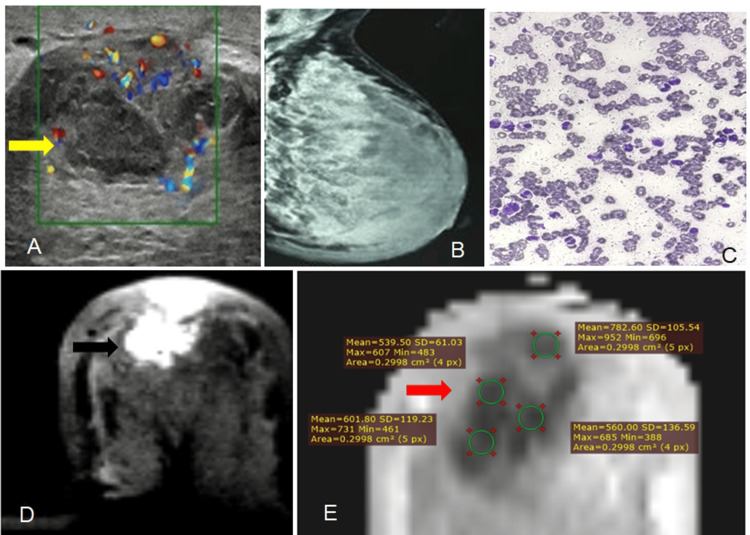
Mucinous Carcinoma (A) Ultrasound shows a heterogeneous, irregular lesion (white arrow) with indistinct margins and intralesional calcifications, categorized as BI-RADS IVc. (B) Mammogram (CC view) reveals an irregular, equal-density lesion (black arrow) in the inner quadrant with indistinct margins and coarse calcifications, also assessed as BI-RADS IVc.(C) Histopathology confirms mucinous carcinoma on photomicrograph. (D) Axial DWI demonstrates a high signal lesion (yellow arrow). (E) The corresponding ADC map (b = 1000) shows a high ADC value (blue arrow), comparable to that of benign lesions; [ADC = 1.2 × 10⁻³ mm²/s].

The present study included 34 lesions in 32 female patients; the age ranged between 35 and 62 years, with a mean age of 43 years old. The commonest finding was the presence of a palpable lump in the breast in 30 cases (88.2%), followed by 12 cases (35.3%) of pain. The majority of the patients were in the age group 35-44 years, i.e., 19 out of 32 patients (59.4%). 16 (47.1%) benign lesions and 18 (52.9%) malignant lesions. Fibroadenoma was the most common benign lesion, accounting for 17.6%. Among the benign lesions, fibroadenomas exhibited the highest mean ADC value (2.03 ± 0.8 × 10⁻³ mm²/s) (Figure 1), while focal mastitis showed the lowest ADC value in the benign group (0.503 ± 0.05 × 10⁻³ mm²/s), mimicking malignancy (Figure 2). Infiltrating ductal carcinoma (IDC) was the most commonly encountered malignant lesion, accounting for 32.3%. Overall, IDC was the most common lesion (Figure 3). In the malignant group, mucinous carcinoma (Figure 4) showed a high mean ADC value (1.2 ± 0.03 × 10⁻³ mm²/s).

BI-RADS category was assigned after assessing ultrasonography (US) and mammography (MG) findings as per the ACR BI-RADS lexicon, showing the following distribution: category 4c: 41.1%, category 4a: 29.4%, category 4b: 17.7%, and category 5: 11.8%.

Qualitative DWI assessment

All patients underwent diffusion-weighted imaging (DWI) as a problem-solving tool. Lesions were qualitatively assessed based on the presence or absence of diffusion restriction (Table 1).

**Table 1:** Qualitative DWI Findings Values are presented as number of cases (N) with corresponding percentages (%).

Diffusion Restriction	Benign (n = 16)	Malignant (n = 18)	Total (n = 34)
Present (+)	3 (8.8%)	16 (47.1%)	19 (55.9%)
Absent (−)	13 (38.2%)	2 (5.8%)	15 (44.1%)

Out of 34 lesions, 19 (55.9%) showed diffusion restriction, of which 16 were malignant and three were benign. The remaining 15 lesions (44.1%) showed no diffusion restriction, among which two were malignant.

Quantitative DWI analysis

The best cut-off ADC value was found to be 1.12 × 10⁻³ mm²/s using ROC curve analysis, with an area under the curve (AUC) of 0.952 (95% CI: 0.891-1.032). Using b-values of 500, 800, and 1000 s/mm², the results were as follows: Mean ADC of benign lesions: 1.51 ± 0.22 × 10⁻³ mm²/s; mean ADC of malignant lesions: 1.00 ± 0.09 × 10⁻³ mm²/s; mean relative ADC (rADC) of benign lesions: 1.02 ± 0.18 × 10⁻³ mm²/s; mean rADC of malignant lesions: 0.67 ± 0.11 × 10⁻³ mm²/s.

There was a highly significant difference in both mean ADC and mean rADC values between benign and malignant lesions (p < 0.0001 and p = 0.0002, respectively; Student’s t-test). The range of mean and relative ADC values of benign and malignant lesions is summarized in Table 2.

**Table 2:** Mean ADC and Relative ADC Values of the Present Study A p-value of <0.05 was considered statistically significant. Statistically significant values are marked with an asterisk (*) ADC: Apparent Diffusion Coefficient

Lesions	n	mADC±SD x10^-3^ mm^2^/s	Range of mADC	rADC±SD x 10^-3^ mm^2^/s	Range of rADC
Benign	16	1.5±0.22	1.2-2.2	1.02±0.18	0.9-1.3
Malignant	18	1.0±0.09	0.8-1.2	0.67±0.11	0.5-0.85
p-value		<0.0001*		0.0002*	

## Discussion

Our findings align with those of Parsian et al. [6], who observed that fibroadenomas with a predominantly fibrous stromal component can show low ADC values. Fibroadenomas demonstrated the highest ADC value among benign lesions (2.03 ± 0.8 × 10⁻³ mm²/s), whereas focal mastitis had the lowest (0.503 ± 0.05 × 10⁻³ mm²/s), which can resemble malignant pathology on DWI. This observation is supported by Woodhams et al., Lee et al., and Canverenler et al., who reported that restricted diffusion may occur not only in highly cellular malignant lesions but also in benign conditions such as abscesses and mastitis due to high viscosity [3,4,7].

Mucinous carcinoma showed a high mean ADC value (1.2 ± 0.03 × 10⁻³ mm²/s), which is consistent with findings by Woodhams et al. [3], who noted that the low cellularity and mucin-rich content of mucinous carcinomas can result in elevated ADC values. Baltzer et al. [8] similarly noted that mucinous carcinomas may be missed by non-contrast DWI protocols and that DCE-MRI is helpful for placing ADC ROIs in enhancing areas for accurate assessment. For invasive ductal carcinoma (IDC), the mean ADC value was 0.831 ± 0.13 × 10⁻³ mm²/s. However, one IDC case in our study had a value above the cutoff (1.2 ± 0.15 × 10⁻³ mm²/s), causing a false-negative result. Hirano et al. [9] similarly reported that pathological heterogeneity in IDC can affect ADC measurements.

The mean ADC value of normal breast parenchyma in our study was 1.68 ± 0.02 × 10⁻³ mm²/s (range: 1.3-2.3). A statistically significant difference was found between the ADC values of malignant lesions and normal breast tissue (p < 0.0001). This finding is consistent with studies by Yilmaz et al. and Kim et al., who also reported significantly higher ADC values in normal tissue compared to cancerous lesions [10,11].

Unlike earlier studies, we used a combination of three b-values (500, 800, and 1000 s/mm²). For all individual b-values, ADC measurements consistently showed lower diffusivity in malignant lesions. The highest lesion detection performance was achieved with b = 1000; however, the best diagnostic performance was obtained using the mean ADC derived from all three b-values. Durur-Subashi emphasized the importance of higher b-values (800-1500 s/mm²) to minimize T2 shine-through effects and improve lesion conspicuity by suppressing background parenchymal signal [12]. Pereira et al. [13] confirmed that using multiple b-values provides more accurate ADC calculations. Our findings support this, as summarized in Tables 3, 4, where ADC values and cutoff thresholds from previous studies are compared with the present study.

**Table 3:** ADC Values Encountered in the Previous Studies *Values within “()” denote b-values in sec/mm^2^ ADC: Apparent Diffusion Coefficient

LESION TYPE	Yilmaz et al. [10] (0, 1000)	Sahin et al. [14] (50, 400,800)	Al-Saadi et al. [15]( 0,600)	Present Study (500, 800, 1000)
Mean ADC range (Malignant)	0.830-1.490	0.52-1.2	0.65-1.46	0.8-1.2
Mean ADC range (benign)	0.833-2.460	1.14-2.66	1.1-1.86	1.2 -2.2
mADC (malignant)	0.884	0.86	0.93	1.0±0.09
mADC (benign)	1.58	1.9	1.5	1.5±0.22

**Table 4:** ADC Cutoff Values Across Studies *Values within “( )” denotes b-values in sec/mm^2^ ADC: Apparent Diffusion Coefficient

	Yilmaz et al. [10]( 0, 1000)	Sahin et al. [14]( 50, 400,800)	Al-Saadi et al. [15] (0,600)	Maaly et al. [16] (1000)	Present Study (500, 800, 1000)
ADC Cut off	1.04	1.03	1.17	1.03	1.12

The diagnostic performance of DWI in our study yielded a sensitivity of 88.8%, specificity of 81.2%, positive predictive value (PPV) of 84.2%, negative predictive value (NPV) of 86.6%, and overall accuracy of 85.3%. A comparison of previous DWI-based studies conducted using 1.5T MRI is shown in Table 5. The variation in sensitivity, specificity, PPV, NPV, and cutoff values among studies may be attributed to the choice of b-values, differences in hardware, lack of standardized imaging protocols, and heterogeneous patient populations.

**Table 5:** Previous Studies Using Non-Contrast MRI Done in 1.5 T MRI (Adapted From: Baltzer et al.) [8] ssEPI: single-shot echo-planar imaging; STIR: short tau inversion recovery; SPAIR: spectral attenuated inversion recovery; DWIBS: diffusion-weighted whole-body imaging with background signal suppression; TSE: turbo spin echo; FLASH: fast low angle shot; FS: fat-suppressed.

	Cancer prevalence (%)	Non contrast sequences	Study population	Sensitivity	Specificity
Baltzer et al.2010 [8]	66.6	ssEPI+T2w-TSE	BI-RADS 4 and5	94.4%	85.2%
Yabuuchi et al. 2011 [17]	66.6	ssEPI+T2wSTIR and SPAIR	Mixed: asymptomatic breast cancers and control group	50.0%	95.2%
Bickelhaupt et al,2016 [18]	48.0	DWIBS MIP+T2w TSE and SPAIR	Screening detected BI-RADS 4 and 5 lesions	91.7%	96.2%
Belli et al. 2016 [19]	44.6	ssEPI+STIR	Mixed: histologically proven cancers and equivocal cases	78.8%	96.9%
Present study	52.9	ssEPI+STIR + non FS T1 FLASH	BIRADS 4 and 5	88.8%	81.2%

Limitations

Several limitations of this study should be acknowledged. First, the sample size was relatively small, which may affect generalizability. Second, there was a lack of standardized acquisition protocols across studies, including variation in b-values and post-processing methods. Third, all imaging was performed on a 1.5T MRI scanner; therefore, our results may not directly apply to higher field strengths such as 3T MRI.

## Conclusions

The goal of all breast imaging modalities is early and accurate detection of malignancy, minimizing unnecessary interventions, and improving patient outcomes. In our study, diffusion-weighted imaging (DWI) with multiple b-values and quantitative apparent diffusion coefficient (ADC) mapping demonstrated good diagnostic performance in differentiating benign from malignant breast lesions. Mean ADC values derived from multiple b-values (b = 500, 800, and 1000 s/mm²) yielded sensitivity and specificity comparable to individual b-values, reinforcing the value of multiparametric DWI in lesion characterization. However, limitations were observed. ADC values can overlap between benign and malignant lesions, potentially leading to false positives and false negatives. Additionally, DWI has limited utility in subcentimetric lesions, cystic lesions, and in identifying non-mass enhancement types such as ductal carcinoma in situ (DCIS) and invasive lobular carcinoma (ILC). Thus, DWI cannot be used as a stand-alone diagnostic tool. Despite these limitations, our findings suggest that DWI with ADC mapping is a fast, noninvasive, and cost-effective adjunct to conventional imaging modalities such as mammography and ultrasound. Particularly in patients unable to undergo contrast-enhanced MRI, such as those with renal impairment or prior contrast reactions, DWI combined with unenhanced sequences may serve as a viable alternative.

These results support the integration of DWI into routine breast imaging protocols and highlight the need for larger, multicenter studies with standardized acquisition protocols. With further validation, DWI has the potential to be incorporated into the ACR BI-RADS lexicon as an unenhanced supplemental screening tool, particularly for high-risk populations.
